# Stimulation of paralysed quadriceps muscles with sequentially and spatially distributed electrodes during dynamic knee extension

**DOI:** 10.1186/s12984-018-0471-y

**Published:** 2019-01-07

**Authors:** Marco Laubacher, Efe A. Aksoez, Anne K. Brust, Michael Baumberger, Robert Riener, Stuart Binder-Macleod, Kenneth J. Hunt

**Affiliations:** 1 Institute for Rehabilitation and Performance Technology, Division of Mechanical Engineering, Department of Engineering and Information Technology, Bern University of Applied Sciences, Pestalozzistrasse 20, Burgdorf, 3400 Switzerland; 2 Sensory Motor Systems Lab, Department of Health Sciences and Technology, ETH Zurich, Zurich, 8000 Switzerland; 3 Swiss Paraplegic Centre, Nottwil, 6207 Switzerland; 4 Department of Physical Therapy, University of Delaware, Newark, United States of America

**Keywords:** Functional electrical stimulation, Spinal cord injury, Rehabilitation, Spatially distributed sequential stimulation, Knee dynamometer, Power output, Fatigue

## Abstract

**Background:**

During functional electrical stimulation (FES) tasks with able-bodied (AB) participants, spatially distributed sequential stimulation (SDSS) has demonstrated substantial improvements in power output and fatigue properties compared to conventional single electrode stimulation (SES). The aim of this study was to compare the properties of SDSS and SES in participants with spinal cord injury (SCI) in a dynamic isokinetic knee extension task simulating knee movement during recumbent cycling.

**Method:**

Using a case-series design, m. vastus lateralis and medialis of four participants with motor and sensory complete SCI (AIS A) were stimulated for 6 min on both legs with both electrode setups. With SES, target muscles were stimulated by a pair of electrodes. In SDSS, the distal electrodes were replaced by four small electrodes giving the same overall stimulation frequency and having the same total surface area. Torque was measured during knee extension by a dynamometer at an angular velocity of 110 deg/s. Mean power of the left and right sides (*P*_*meanL*,*R*_) was calculated from all stimulated extensions for initial, final and all extensions. Fatigue is presented as an index value with respect to initial power from 1 to 0, whereby 1 means no fatigue.

**Results:**

SDSS showed higher *P*_*meanL*,*R*_ values for all four participants for all extensions (increases of 132% in participant P1, 100% in P2, 36% in P3 and 18% in P4 compared to SES) and for the initial phase (increases of 84%, 59%, 66%, and 16%, respectively). Fatigue resistance was better with SDSS for P1, P2 and P4 but worse for P3 (0.47 vs 0.35, 0.63 vs 0.49, 0.90 vs 0.82 and 0.59 vs 0.77, respectively).

**Conclusion:**

Consistently higher *P*_*meanL*,*R*_ was observed for all four participants for initial and overall contractions using SDSS. This supports findings from previous studies with AB participants. Fatigue properties were better in three of the four participants. The lower fatigue resistance with SDSS in one participant may be explained by a very low muscle activation level in this case. Further investigation in a larger cohort is warranted.

## Background

Mobilisation and exercise play an important role during the rehabilitation of persons after a spinal cord injury (SCI). On the one hand it is important to regain strength and mobility, and on the other hand it helps to prevent secondary complications [1–3]. Arm cycle ergometry or long wheelchair runs are often used for cardiovascular training and to maintain fitness [4], but these exercise modes only include upper body movement and the paralysed leg muscles remain inactive. As a result, blood pooling may occur in the lower limbs, and low blood pressure and deep venous thrombosis are possible consequences [5, 6]. Functional electrical stimulation (FES) provides a means of mobilising intact lower leg structures and has been shown to be very effective in preventing these secondary complications when used regularly over a period of time [7–9]. Positive adaptations of bone mineral density and increased muscle strength are further benefits following electrical stimulation training [10, 11]. Although these improvements help to reduce the risk of pressure sores and fractures, the application of FES is not yet fully integrated into the rehabilitation following severe spinal cord injury because the costs can be high and the health benefits may not be immediately apparent [12]. FES training is often replaced with other rehabilitation programmes or restricted to patients with incomplete lesions where motor learning and carry over effects are expected and observed [13].

Two major factors that limit the use and effectiveness of FES are the well-known problems of rapid muscle fatigue and low power output of artificial stimulation when used for functional tasks [14–18]. In the last 40 years, different approaches have been used to address these challenges. Pulse modulation, electrode placement or diversifications of electrodes are strategies applied to increase power or fatigue resistance. Most effort was placed on investigating the effects of pulse modulation, such as changing stimulation frequency, pulse width or amplitude. Constant, low frequency trains of pulses show better fatigue resistance compared to high frequency trains, but on the other hand the power output is significantly lower [19–22]. Adding doublets to stimulation trains increased the fatigue resistance and force production significantly, especially when applied to fatigued muscles [23–25]. Stochastic inter-pulse intervals showed inconsistent results. Depending on the specific task, fatigue resistance and power output were in some cases increased and in other cases worse compared to traditional stimulation patterns [26–29].

Stimulation intensity can also be controlled using pulse width or amplitude. High intensity is usually correlated to high power output [15, 30]but entails decreased fatigue resistance [31–33]. The increases in power, observed when stimulation intensity is increased, is related to a higher number of activated motor units, but the precise recruitment mechanisms are unclear. It is proposed that the enlarged cross sectional activation area with higher pulse widths results from increased electrical signal propagation within the muscles, while increased amplitude will increase the current density and the electrical field will reach deeper structures [31, 34, 35]. Since it has been observed that higher intensities can cause more antidromic impulses [36], investigations in modulating the stimulation amplitude often include changes in the pulse width to keep current and stimulation intensity constant.

Most investigations have shown some improvements in either power or fatigue but no study to date has demonstrated meaningful increases in both power and fatigue. Non-selective recruitment of motor units and poor intra- and inter-muscular coordination leads to an exaggerated metabolic cost of electrically evoked contractions [16, 17]. This problem also exists in able-bodied persons, where power drops 30% shortly after stimulation onset (compared to 50% fatigue in SCI) [37]. This indicates that, in part, low power output and high fatigue result from the characteristics of artificial muscle stimulation. Surface electrodes are spatially fixed and the underlying motor units are activated synchronously as soon as the critical threshold is reached. Since the location of the electrodes is a crucial part of the application, even small changes in placement can change the power substantially [38–40].

The use of more electrodes and spatial differentiation, termed spatially distributed sequential stimulation (SDSS), has been shown to be very effective in power generation and fatigue reduction [41–43]. With distributed electrodes, the electrical field can be varied and more motor units will be activated, thus more power can be generated [44]. To date, several different strategies have been investigated. Placing the electrodes on synergistic muscles will produce more power by increasing the activated muscle mass [9, 42, 43], while placing more electrodes on the same muscle belly will increase the number of activated fibres of the same muscle [44–47]. The synchronous activation of all motor units below the electrodes is one major issue in the poor fatigue resistance with FES. With distributed electrodes, it is possible to add a temporal shift between the pulses and decrease the stimulation frequency for each electrode, while maintaining the same overall stimulation frequency. This setup, with the alternated activation of more motor units, has been shown to increase fatigue resistance significantly in several studies and to maintain or even increase power output compared to conventional ES [44–47]. The lower stimulation frequency applied to each electrode allows for a lower ATP cost for each contraction and is more efficient in binding cross-bridges. It is believed that the lower frequency causes fewer problems in Ca2+ release than are observed with high frequency stimulation. Furthermore, the mechanism whereby the electric field is changed constantly might activate other neural circuits, which again activates some other muscle parts in the same muscle group [44, 48, 49].

The increasing number of studies with positive outcomes regarding distributed electrode setups indicates that this strategy is a promising solution for the low power and the low fatigue resistance of conventional surface electrode stimulation. However, the number of studies with SCI participants is limited and the transfer of knowledge gained with able-bodied participants to SCI is another challenge [25]. Following an SCI there is a rapid loss of muscle mass and a change in muscle nerve physiology based on histological changes in muscle composition. Muscle atrophy is very specific in each muscle depending on fibre type, body composition and training status before injury. A decrease in muscle cross sectional area of 20 – 50% 6 months after injury is usual for unloaded muscles and comparable to long bed rest or space flight in able-bodied persons. The histochemical changes take longer and become significant 6 to 12 months after injury. Thus, the proportional distribution of fast- and slow-twitch fibres does not change significantly during the first weeks after injury [50–52]. Clearly, these changes in physiological structure influence the interaction between electrode and muscle [53]. With non-isometric FES, the physical movement of muscle bulk is another factor affecting tissue resistance. This can induce the activation of other nerve fibres and reflexes, which can then disturb the functional movement. As a consequence, for power or fatigue related investigations with surface electrodes, it is very important to mimic the intended task as precisely as possible [54, 55].

The aim of this study is to compare the power output and fatigue properties of the quadricps femoris muscles in response to spatially distributed sequential stimulation (SDSS) versus traditional single electrode stimulation (SES) in four untrained participants with motor-complete spinal cord injury during a dynamic leg extension task simulating knee joint movement in recumbent cycling.

## Method

Four participants with motor-complete spinal cord injury and an American Spinal Cord Injury Association (ASIA) impairment score (AIS) A were included (Table 1) according to a case series study design [56]. Each participant gave written informed consent. The study was approved by the local ethics committee (Ethics Committee of northwest/central Switzerland, Ref.-Nr: BASEC 2016-00394).

**Table 1:** Characteristics of the four participants

Participant	Sex	Age (yrs)	Height (cm)	Body mass (kg)	Time since injury (months)	Lesion level	Rehab-status
P1	f	45.4	168	61.3	23.8	T3	Re-Reha
P2	m	22.9	187	80.0	5.3	T6	First-Reha
P3	m	48.4	178	75.0	5.8	T2	First-Reha
P4	m	27.5	183	65.0	5.6	T5	First-Reha
mean ± sd		36.1 ± 12.7	179 ± 8.2	70.3 ± 8.7	10.1 ± 7.9		

First-Reha: Primary rehabilitation phase directly after injury;

Re-Reha: Second entry into the rehabilitation clinic, after being at home following primary rehabilitation

### Measurement instruments

Measurements were conducted using an isokinetic dynamometer (Cybex IIa, Biodex Medical Systems Inc., USA) with an upgrade package (Humac Norm, Computer Sports Medicine Inc., USA). The dynamometer was set up for concentric quadriceps measurement. After seating, individual adjustments were made for ergonomic knee joint movement.

The dynamometer controlled the angular range of motion at the knee joint from 40 to 120 deg (180 deg means straight leg) at an angular velocity of 110 deg/s at the knee joint, which is equivalent to a cycling cadence of 50 rpm. The dynamometer was interfaced to a PC running Matlab/Simulink and the Real-Time Toolbox (MathWorks Inc., USA), which recorded raw data of the knee angle, torque and time. These data were used to control the stimulation range and the stimulation parameters. A graphical user interface was implemented for setting up and controlling the stimulation device and the timing (Aksoz EA, Laubacher M, Binder-Macleod S, Hunt KJ, Design of an isokinetic knee dynamometer for evaluation of functional electrical stimulation strategies, submitted).

### Stimulation

**Fig. 1:**
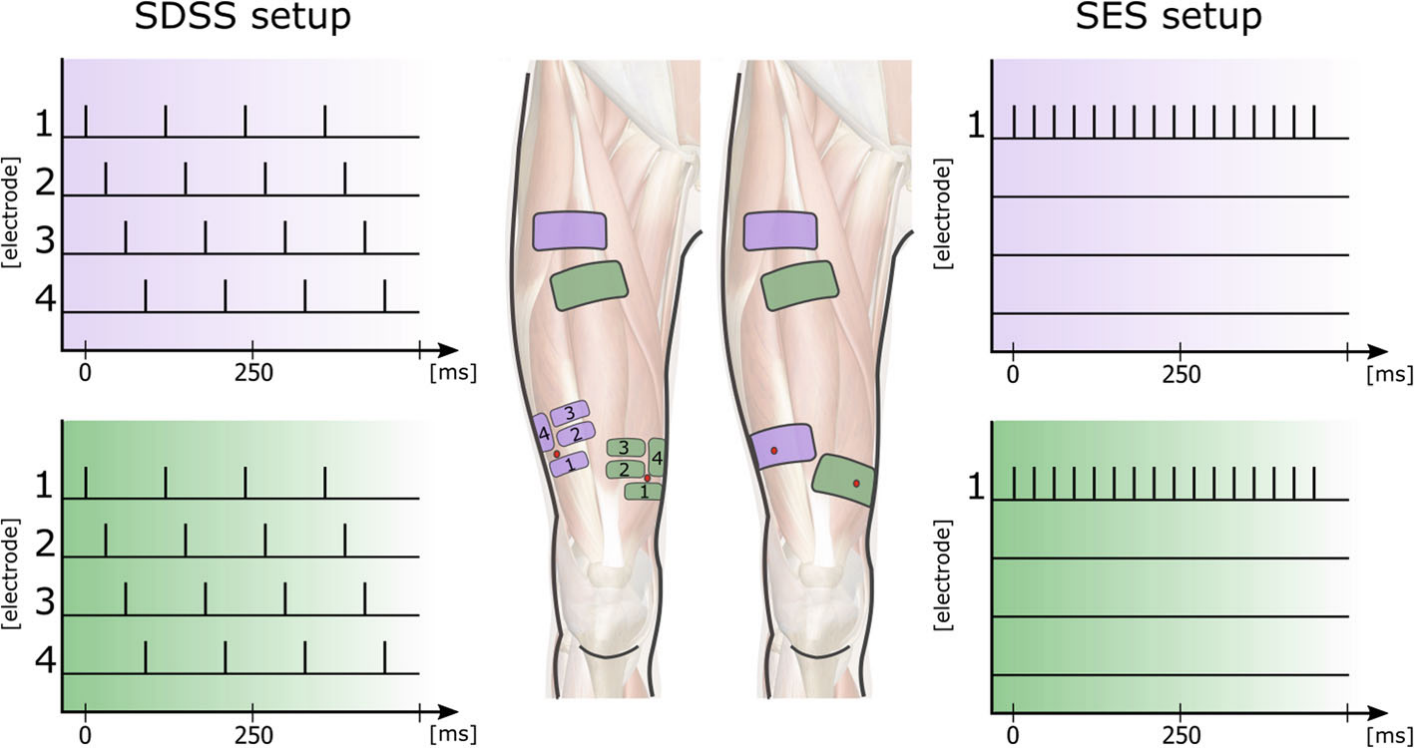
Schematic picture of the electrode setup and the corresponding stimulation pattern. The purple and green electrodes are synchronised and stimulate synergistically the m. lateralis (purple) and m. medialis (green). Visible on the left: the SDSS setup with four small electrodes replacing the active electrodes. Electrodes were placed as close as possible to the located motor points. SES setup with two pairs of electrodes on the right side. Active electrodes were placed on the motor points of m. vastus medialis and m. vastus lateralis. Motor points are depicted with a red dot

Before measurement, the skin was cleaned and the body hair shaved at the position of the electrodes. For each stimulated muscle, one motor point was detected prior to measurement with a stimulation pen (Motor Point Pen, Compex SA, Switzerland). Motor points and electrode positions were marked to ensure identical placement across the sessions. The investigated stimulation setup was similar to a previous study with able-bodied participants [44]. Two different electrode setups were compared: SES and SDSS. In both setups, participants were stimulated with rectangular bi-phasic pulses of constant 40 mA amplitude generated under PC control with an eight-channel stimulator (RehaStim, Hasomed GmbH, Germany) with a pulse width range of 0 - 500 *μ*s (1 *μ*s steps) and a frequency range of 0 - 100 Hz. For the SES setup, a single pair of self-adhesive electrodes with a dimension of 9 × 5 cm (Pals Platinum, Axelgaard Mfg. Co., LTD, USA) were placed on the motor points of the m. vastus lateralis and medialis. Reference electrodes with the same size were placed 10 - 15 cm proximal of the corresponding muscle motor point and the frequency was set at 35 Hz. In the SDSS setup, four small electrodes each with a size of 4.5 × 2.5 cm were placed around the previously detected motor point. Each of the four electrodes was stimulated with a frequency of 8.75 Hz and a phase shift of 90 deg, which corresponds to the stimulation frequency of the SES setup of 35 Hz and using the same total electrode area. The SDSS stimulation order was always the same, i.e. from 1 to 4, and the reference electrodes were the same as for the SES setup (Fig. 1). For both setups, stimulation was applied only during the knee-extension phase of the motion, over a knee-angle range of 55 deg to 115 deg. In each session the pulse width was adapted to the participant according the familiarization procedure detailed below. For this study, the mean pulse width applied was 213.4 ± 41.2 *μ*s for SES and 217.8 ± 35.8 *μ*s for SDSS.

### Procedure

Measurements were conducted in two sessions with at least 24 h between each session. Within each session, each leg was stimulated with only one electrode configuration. Between the two independent leg measurements participants had a break of 15 min. Stimulation and leg order (SES then SDSS vs. SDSS then SES; L then R vs. R then L) was chosen randomly. Motor point detection was conducted prior to the first measurement while participants were seated in their wheelchair. Participants where then transferred to the dynamometer system and individual adjustments to body proportions were made. Before each stimulation (leg and setup), a brief familiarization was conducted to determine stimulation parameters and to control the device setup.

The familiarisation started with a two-minute passive phase in which the measured leg was moved by the device without stimulation (non-stimulation phase, ns-phase). The data collected during this phase was used as a baseline measurement for the leg movement resistance. The next phase involved stimulation while the leg was being moved by the dynamometer. The pulse width was initially set at 45 *μ*s and was manually increased after every extension until the power output started to plateau. Stimulation was halted and the pulse width needed to produce the maximum power (*PW*_*max*_) was noted. The pulse width used for the subsequent measurement was 80% of *PW*_*max*_ (*PW*_*m*80_=0.8∗*PW*_*max*_). After a 10-min rest period following familiarisation, the actual measurement was commenced with an ns-phase of two min and a stimulation phase (st-phase) of six min. A second two min ns-phase completed the measurement.

### Outcomes and data analysis

The measured torque together with the angular speed was used to calculate the gross power output *P*_*m*_ generated during the stimulated knee extension. The power used to move the leg during the ns-phase was defined as *P*_*ns*_ and the net power output of one stimulation cycle, *P*_*stim*_, was then obtained as *P*_*stim*_=*P*_*m*_−*P*_*ns*_. The mean power output over the stimulation angle range during one extension (*P*_*mean*_), peak power output (*P*_*peak*_) and the time from onset of the stimulation to 80% of *P*_*peak*_ (*t*_*peak*80_) was calculated for each extension. For each of these three output parameters mean values and standard deviations were calculated for the initial 10 stimulated extensions (init), the final 20 extensions (final) and all 160 extenstions (overall) for each of the four subjects. *P*_*meanL*,*R*_ is denoted as the mean power output of the left and right legs.

A fatigue index (FI) based on *P*_*mean*_ describes the loss of power between the ten initial knee extensions (*P*_*init*_) and the final 20 knee extensions (*P*_*final*_) from the stimulated phase. Thus, *FI*=1−(*P*_*init*_−*P*_*final*_)/*P*_*init*_. The higher the value, the higher the fatigue resistance; *FI*=1 means no fatigue.

## Results

### Overall outcomes

**Fig. 2:**
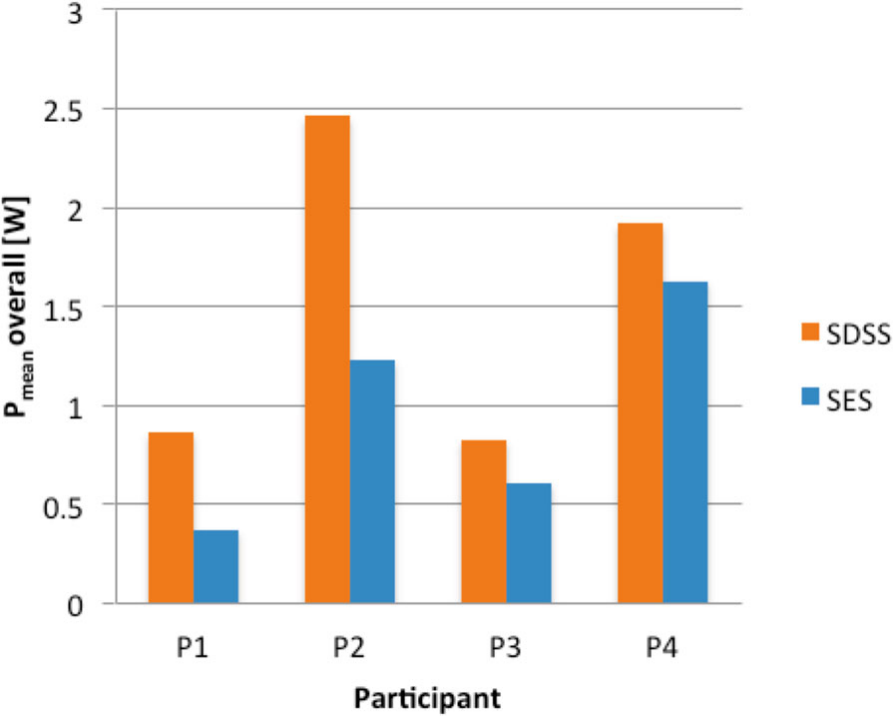
Mean power response to all 160 extension measurements (*P*_*meanL*,*R*_ overall) for each participant

The SDSS setup gave higher *P*_*meanL*,*R*_ values for all four participants overall (increases of 132% in P1, 100% in P2, 36% in P3 and 18% in P4 compared to SES, Fig. 2) and for the initial phase (increases of 84%, 59%, 66% and 16%). Fatigue resistance was better with SDSS for P1, P2, P4 but worse for P3 (0.47 vs 0.35, 0.63 vs 0.49, 0.90 vs 0.82 and 0.59 vs 0.77, respectively). No valid values were available for *P*_*peak*80_ and *t*_80_ since not every stimulated extension had a clear peak.

**Fig. 3:**
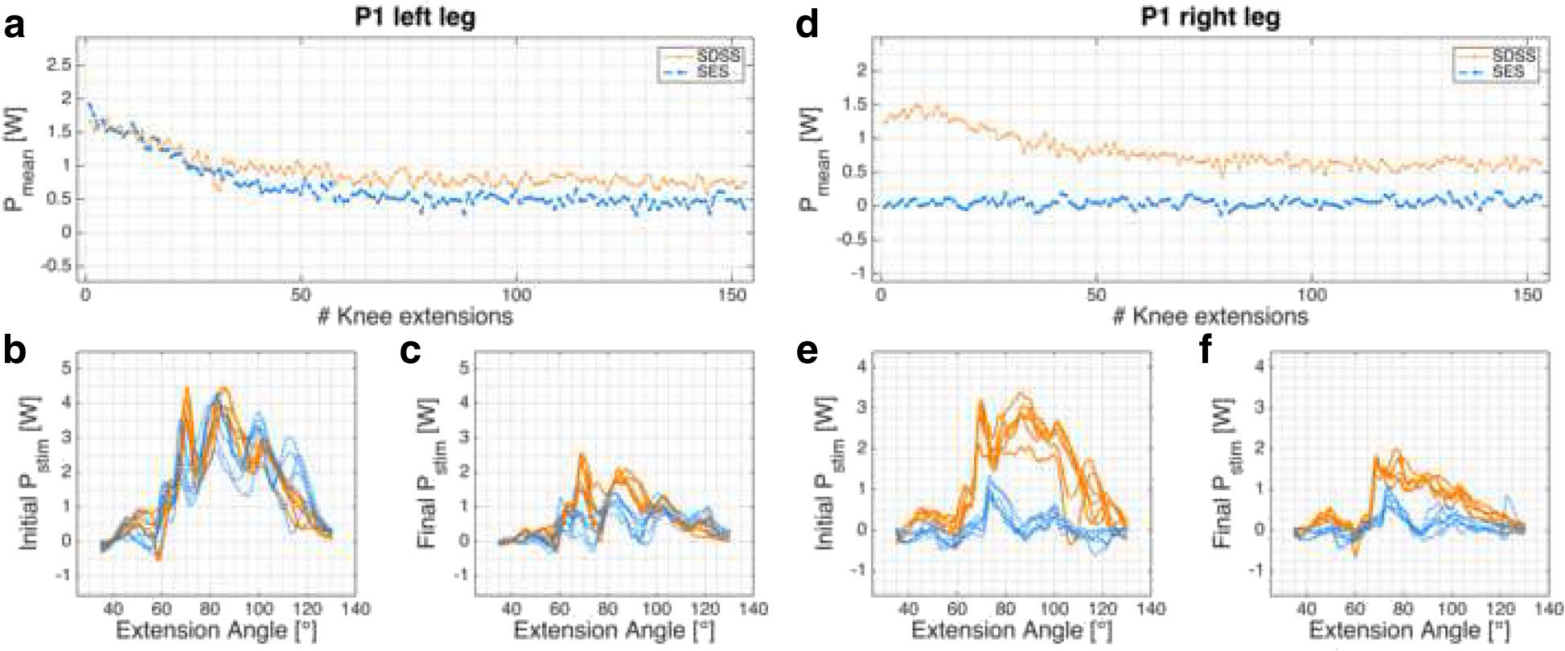
P1 power profile. *P*_*mean*_ of P1’s left (**a**)-(**c**) and right (**d**)-(**f**) legs. **a**, **d**: progression of *P*_*mean*_ over the 6-min knee extension task. **c**, **d**, **e**, **f**: power profile *P*_*stim*_ for each leg and setup in the initial and final phases

**Fig. 4:**
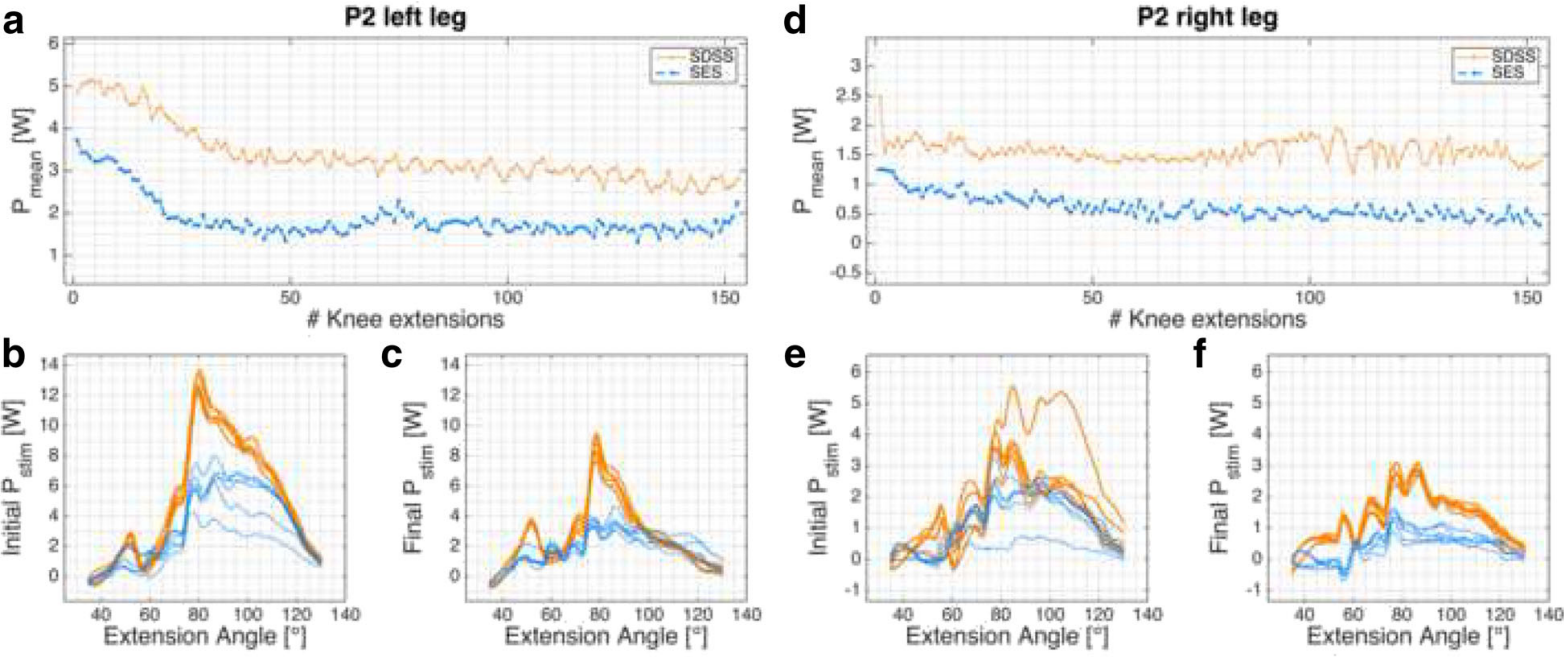
P2 power profile. *P*_*mean*_ of P2’s left (**a**)-(**c**) and right (**d**)-(**f**) legs. **a**, **d**: progression of *P*_*mean*_ over the 6-min knee extension task. **c**, **d**, **e**, **f**: power profile *P*_*stim*_ for each leg and setup in the initial and final phases

**Fig. 5:**
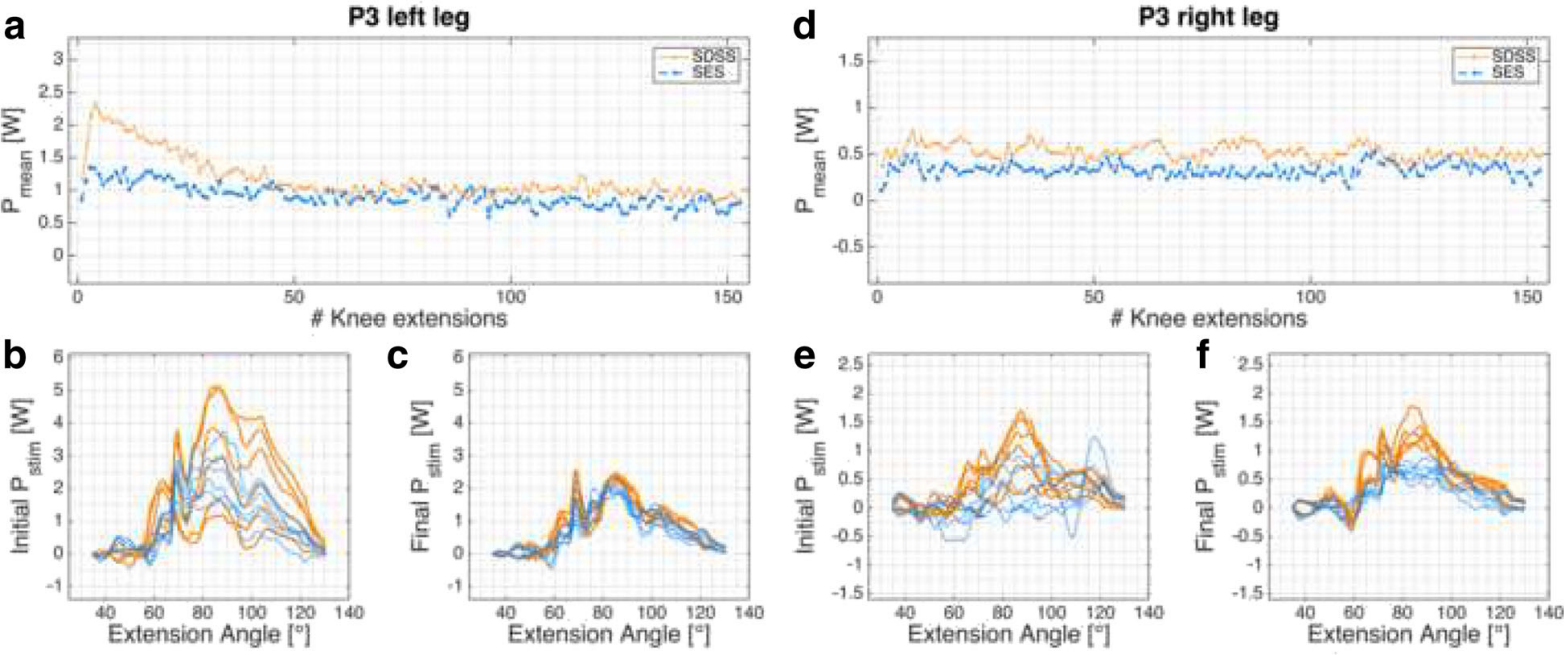
P3 power profile. *P*_*mean*_ of P3’s left (**a**)-(**c**) and right (**d**)-(**f**) legs. **a**, **d**: progression of *P*_*mean*_ over the 6-min knee extension task. **c**, **d**, **e**, **f**: power profile *P*_*stim*_ for each leg and setup in the initial and final phases

**Fig. 6:**
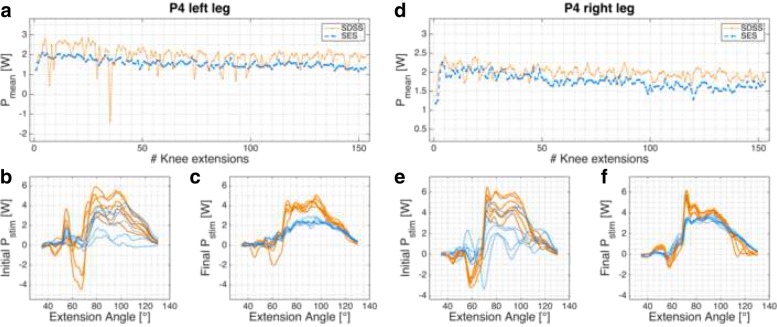
P4 power profile. *P*_*mean*_ of P4’s left (**a**)-(**c**) and right (**d**)-(**f**) legs. **a**, **d**: progression of *P*_*mean*_ over the 6-min knee extension task. **c**, **d**, **e**, **f**: power profile *P*_*stim*_ for each leg and setup in the initial and final phases

The results are summarized in Table 2 and the individual power development of each leg is shown in Figs. 3, 4, 5 and 6.

**Table 2:** Power output, fatigue values and corresponding pulse width of each measurement

		Pmean [W]						Fatigue		Pulse width [ *μs*]	
		Initial		Final		Overall		Index			
Participant	Leg	SDSS	SES	SDSS	SES	SDSS	SES	SDSS	SES	SDSS	SES
P1	L	1.54	1.53	0.74	0.47	0.93	0.69	0.48	0.31	176	196
	R	1.37	0.05	0.61	0.08	0.80	0.05	0.45	1.69	188	128
	meanL,R	1.45	0.79	0.68	0.27	0.86	0.37	0.47	0.35	182	162
	% diff	84%		147%		132%		34%		12%	
P2	L	4.92	3.18	2.66	1.60	3.37	1.86	0.54	0.50	185	195
	R	1.75	1.01	1.52	0.44	1.56	0.60	0.87	0.44	180	192
	meanL,R	3.33	2.10	2.09	1.02	2.46	1.23	0.63	0.49	182.5	193.5
	% diff	59%		104%		100%		28%		-5%	
P3	L	1.87	1.14	0.94	0.77	1.14	0.90	0.50	0.67	240	250
	R	0.55	0.31	0.48	0.34	0.51	0.32	0.88	1.11	250	240
	meanL,R	1.21	0.73	0.71	0.56	0.83	0.61	0.59	0.77	245	245
	% diff	66%		27%		36%		-23%		0%	
P4	L	2.04	1.78	1.87	1.37	1.88	1.53	0.92	0.77	260	256
	R	2.13	1.83	1.89	1.61	1.96	1.73	0.89	0.88	260	250
	meanL,R	2.08	1.81	1.88	1.49	1.92	1.63	0.90	0.82	260	253
	% diff	16%		26%		18%		10%		-3%	

***% diff:*** *Percentage difference between SDSS > SES for parameter meanL,R*

***initial:*** *first 10 extensions;* ***final:*** *last 20 extensions*

### Individual cases

#### Case 1

Participant P1 (data records in Fig. 3) had lesion level T3, AIS A, and markedly atrophied leg muscles. The measurements were conducted 24 months post injury at the second entry in the clinic (re-rehabilitation). This participant had muscle contractions in the abdominal region during the familiarization session (SES setup, right leg). Co-contractions of m. sartorius and adductor muscles were visible during familiarization and small spastic leg activations were observed at the beginning of each measurement. The left leg showed only minor differences between the two stimulation setups, whereby the SDSS had a higher fatigue resistance (0.47 for SDSS vs. 0.35 for SES).

#### Case 2

Participant P2 (Fig. 4) had lesion level T6, AIS A, and the injury happened 5 months before the measurement, which took place in the last week of primary rehabilitation. *P*_*mean*_ (overall) produced with the left leg was doubled for both setups compared to the output of the right leg (Table 2). This participant showed co-contractions in the hamstring muscles in all four measurements. The strongest co-contractions were observed during the first 2 min of the SDSS measurement in the right leg but they steadily decreased. Motor points were successfully detected in all stimulated muscles.

#### Case 3

Participant P3 (Fig. 5) had lesion level T2, AIS A, and the measurement took place 6 months after injury during primary rehabilitation. No co-contractions and very low muscle tone were observed. Motor points were only detected for the m. vastus medialis on both legs. On both m. vastus lateralis, the electrodes had to be placed based on guidelines from the literature and on experience.

#### Case 4

Participant P4 (Fig. 6) had lesion level T5, AIS A, and the measurement took place 6 months after injury during primary rehabilitation. Strong co-contractions in the hamstring muscles were observed in all four measurements, mainly at the beginning and then decreasing. This participant showed high muscle tone and the muscles twitched already when cold electrodes were being applied on the skin.

## Discussion

The aim of this study was to compare the power output and fatigue properties of the quadricps femoris muscles in response to spatially distributed sequential stimulation (SDSS) versus traditional single electrode stimulation (SES) during a dynamic leg extension task in four untrained participants with motor-complete spinal cord injury. The task was designed to simulate knee joint movement in recumbent cycling for future applications with FES.

In all four participants the mean power output during stimulated knee extension was higher with the SDSS setup. This outcome is in line with previous measurements with able-bodied participants with the same measurement protocol [44]. Three out of four participants showed higher fatigue resistance with the SDSS setup. However, the FI outcome is not as consistent as *P*_*mean*_. These results suggests that SDSS is beneficial overall when compared to SES and that it may be beneficial in a wide range of FES applications. The following discussion analyses the differences between SDSS and SES in detail.

### Power output

Compared to previous measurements with able-bodied participants, the power output reached in this study was very low and likely not sufficient to perform functional tasks. All four participants were using FES for the first time and their muscles were untrained and already atrophied due to reduced muscle activity following the spinal cord injury. After such an injury, morphological and contractile changes occur in the muscles below the level of the lesion. A decrease in muscle cross-sectional area and reduced enzymatic activity leads to low muscle power as observed in participants P1 and P3 [50, 57, 58]. The measured power corresponds to the observed muscle tone and the time since injury. Participant P1, paralysed for almost two years, reported spastic reflexes, which might have preserved some muscle structure [30, 59]. Participant P3, in contrast, was paralysed for six months but showed very low muscle tone and motor points were only detected for vastus medialis on both legs. Another point often neglected in measuring spinal cord injured participants is the history before injury. Eser et al. [22] concluded that activity level before injury and thickness of the participant’s fat layer is strongly related to the power output achieved by FES. Here, participants P2 and P4 were both aged between 20 and 30 years and reported being regularly physically active before injury. Participants P1 and P3 were aged between 40 and 50 years and reported no specific regular activities before injury. This might further explain the differences seen in power output. Nevertheless, in all four participants the electrical stimulation produced measurable muscle-based power responses. To regain strength and increase muscle cross-sectional area, repeated muscle stimulation over a longer period would be necessary [60–64].

Eight legs of four different participants were measured and in all cases the SDSS setup produced a higher power output compared to the conventional SES setup. With the exception of participant P1’s left leg, the initial power output produced with SDSS was markedly superior to SES. Power is strongly related to the number of motor units recruited, so SDSS is probably able to recruit more motor units in SCI participants [65, 66]. Whether this increase in motor units comes from the larger surface area covered with SDSS, since the electrodes are more distributed than with SES (total electrode size is equal to SES) or through the deeper stimulation by the small electrodes remains unclear [35, 67, 68]. In all power profile plots SDSS produced sharper peaks (Figs. 3b, c, e, f, 4b, c, e, f, 5b, c, e, f, and 6b, c, e, f). Especially in participant P2, the difference in power comes mainly from the sharp peak at 80 deg knee angle (Fig. 4d). This may be due to more contracting fibres in deeper structures, which may be activated through higher current densities produced with SDSS electrodes [69, 70].

### Fatigue

Stimulation intensity is strongly correlated with muscular fatigue, during both voluntary contractions and in artificial muscle stimulation [15, 71]. In this study we increased pulse width during the familiarization process until the increase in force was no longer linearly related to the increased pulse width [63]. This had to be done very quickly, due to the possibility of fatigue onset, which would have influenced the subsequent measurement. This observation was done in real time using the screen of the Cybex device. Compared to the method used with able-bodied participants [44, 68], this method relies only on muscular properties and is not influenced by subjective pain tolerance, which is not applicable in persons with AIS-A SCI.

Three out of four participants showed increased fatigue resistance with SDSS (Table 2). Analysis of individual legs revealed that in two SES measurements final *P*_*mean*_ was higher than initial *P*_*mean*_ (Figs. 3d, 5d). In both cases, the power output was very low and the difference of 0.03 W is marginal. Reducing joint and muscle resistance during the ongoing task is one possible explanation. Here, with SCI participants it is most probably due to reduced muscle tone resulting from FES [72]. On the other hand, both of these measurements gave a very low *P*_*mean*_. Thus, the muscular load as well as the intensity were very low, which is consistent with previous observations that low intensity highly correlates with high fatigue resistance [21, 30, 73].

With regard to progression of knee extension (Figs. 3a, d, 4a, d, 5a, d, and 6a, d), in only six of 16 measurements the progression of the curve showed a rapid decline after 10 - 20 knee extensions, as was seen in able-bodied participants [44]. Five of these six measurements were done with SDSS stimulation; only participant P1’s left leg stimulated with SES showed the same shape of curve. All other measurements have shown a steady decline in *P*_*mean*_ or fatigue index values higher than 0.8. Although a high fatigue index means good fatigue resistance, which is desirable, those high values strongly correlate with low *P*_*mean*_ overall, which is suboptimal but in consistent to other reports in the literature [15, 19, 21, 71, 73–75].

The steady decrease in force might be a sign of recruitment of slow-twitch fibres, whereas a rapid decrease of the initial *P*_*mean*_ might indicate the activation of fast-twitch fibres. Participants P2, P3 and P4 were 6 months post injury and the measurements were conducted around the time at which oxidative enzymatic activity is expected to start to decline and the proportion of fibres that co-express both fast and slow myosin heavy chain isoforms increase [50, 76]. The composition of Type I and Type II fibres should not have changed substantially and might still be around 40/60 for m. lateralis and m. rectus femoris [77]. Based on the review of Biering-Sorensen et al. [76], the measurement with participant P1 was 23 months post injury and therefore during a stable phase of the fibre-type transformation process. However, implicating the recruited muscle fibre type based on these observations is not justified even though the type of muscle fibre recruited is clearly one parameter which influences the progression of the curve.

### Unintended stimulation

In addition to changes in muscle fibre structure and loss of enzymatic activity, the whole body structure, such as fat mass and bone density, changes after a spinal cord injury [57]. The muscle’s physiological properties influence the power and fatigue observed in response to electrical stimulation in a similar way to voluntary muscle contraction in able-bodied persons. In contrast to contractions which are voluntary or via implanted electrodes, muscle activation with surface electrodes has to overcome tissue resistance [67, 70, 78]. With SDSS the same amount of current is applied to a smaller surface area than during SES, which generates a higher current density and it is assumed that the resulting electrical field reaches deeper regions. In a functional task, this resistance is constantly changing due to muscle bulk shift and skin movement. Together with atrophied muscles and increased fat layers, it becomes more challenging to activate target muscles. It can happen that non-targeted nerves are stimulated as observed in participant P1’s right leg. The stimulation was accompanied by uncomfortable contractions in the abdominal region despite the fact that the stimulation intensity was lower compared to the other measurements. Relocating the proximal electrodes during the familiarization did not help to reduce these contractions. Participants P2 and P4 had co-contractions with a short delay to the activated stimulation in all measurements in the hip flexor muscles. These muscles are activated through central pathways, which means the electrical signal was transmitted from sensory nerves via the spinal cord at levels L2/L3 to motor neurons. The co-contractions were strongest with SDSS and gradually decreasing [72]. In addition to hip flexor activity, participant P4 also had antagonistic muscle activity. Best visible as negative power at around 60 deg and in the time plot of the left leg as negative outliers (Fig. 6a, b, e). The gradual decrease of these effects gave relatively high final *P*_*mean*_ values, which, as noted above, explains the very high fatigue resistance in these measurements.

With regard to power output and fatigue properties, this study revealed some major benefits of the SDSS setup compared to SES in participants with motor- and sensory-complete SCI. One limitation of the present study is the case-series design and the concomitant low number of participants. This does not mean that FES is not applicable for other persons with SCI with different impairment classification, but for homogeneity and to reduce confounding factors only persons with motor- and sensory-complete SCI were included. In a next step, the focus should be on recruiting more SCI participants with different grades of injury, and to recruit a sufficient number of participants to allow well-powered statistical analysis. The pilot data from the present study provide a basis for a sample-size and statistical-power calculation.

## Conclusions

This study demonstrated higher power output for the SDSS setup in all eight leg-to-leg comparisons. Fatigue characteristics for the SDSS setup looks promising and SDSS seems to be more resistant when both setups activate the muscle at the same intensity level. Final *P*_*mean*_ was higher for SDSS in all measurements. These outcomes are consistent with the results of previous measurements with able-bodied participants using the same test protocol [44] and demonstrate for the first time the promise of the SDSS approach in untrained people with SCI. Future studies should investigate long-term training protocols and compare SDSS and SES in SCI participants with well-trained muscles. Laubacher et al. [79] reported a single case which compared SDSS and SES in a well-trained person with SCI in the context of preparations for the FES bike race at Cybathlon 2016 in Zurich [80]. In this case, *P*_*meanL*,*R*_ with SDSS was more than twice as high as *P*_*meanL*,*R*_ with SES. These positive results should encourage the development of more sophisticated electrode setups for use in gross motor movements, such as in leg cycling. To date, several studies have shown increased power and fatigue resistance based on multi-electrode setups; combining these approaches with modulation of stimulation parameters might be a good future strategy to further increase power and fatigue resistance [81–83]. A further important aspect for better usability would be to apply multi-electrodes in cuffs or garments with an integrated method to determine the optimal stimulation setup.
